# Structure and Photoluminescence Properties of Sm^3+^ Ion-Doped YInGe_2_O_7_ Phosphor

**DOI:** 10.3390/ma10070779

**Published:** 2017-07-10

**Authors:** Hung-Rung Shih, Yee-Shin Chang

**Affiliations:** 1 Department of Mechanical and Computer-Aided Engineering, National Formosa University, Huwei, Yunlin 632, Taiwan; hrshih@nfu.edu.tw; 2 Department of Electronic Engineering, National Formosa University, Huwei, Yunlin 632, Taiwan

**Keywords:** phosphor, samarium, optical properties, luminescence

## Abstract

A new phosphor, Sm^3+^ ion-doped YInGe_2_O_7_, was synthesized using a planetary ball mill solid state reaction. The XRD patterns show that all of the peaks can be attributed to the monoclinic YInGe_2_O_7_ crystal structure when the Sm^3+^ ion concentration is increased up to 20 mol %. Under an excitation wavelength of 404 nm, the Sm^3+^ intra-4f transition appears in the emission spectrum including two stronger emission peaks located at 560–570 nm and 598 nm correspond to the ^4^G_5/2_ → ^6^H_5/2_ and ^4^G_5/2_ → ^6^H_7/2_ transitions, respectively, and another weak emission peak located at 645 nm is due to the ^4^G_5/2_ → ^6^H_9/2_ transition. The decay time decreases from 4.5 to 0.8 ms as Sm^3+^ ion concentrations increase from 1 to 20 mol %, and the decay mechanism of the ^4^G_5/2_ → ^6^H_7/2_ transition is a single decay component between Sm^3+^ ions only. The concentration quenching effect occurs when the Sm^3+^ ion concentration is higher than 3 mol %. The CIE color coordinate of Y_0.97_Sm_0.03_InGe_2_O_7_ phosphor is at x = 0.457 and y = 0.407, which is located in the orange-yellow light region.

## 1. Introduction

Over the last decade, luminescent properties of inorganic phosphors have been extensively investigated to make flat panel displays such as field emission displays (FEDs), plasma display panels (PDPs), and thin film electro-luminescent devices (TFEL). Many efforts have been made to discover oxide based phosphors to improve luminescent performance—including color purity, emission intensity, and quantum efficiency—because of the higher chemical stability of oxide phosphors relative to that of sulfide phosphors. Many studies have been conducted for developing new oxide phosphors in powder form suitable for full-color emissive displays [1,2,3,4,5,6,7].

Yttrium indium germanate (YInGe_2_O_7_) has the thortveitite structure with symmetry described by the space group C2/m (No. 12). This crystallizes in the monoclinic system, with cell parameters a = 6.8286 Å, b = 8.8836 Å, c = 4.9045 Å. The In^3+^ and Y^3+^ cations occupy the same octahedral site forming a hexagonal arrangement on the a–b planes [8]. In their turn, the hexagonal arrangements of InO_6_/YO_6_ octahedral layers are held together by sheets of isolated diorthogroups composed of a double tetrahedral sharing a common vertex. It is suggested that the YInGe_2_O_7_ possesses excellent optical properties. Previous studies indicated that YInGe_2_O_7_ doped with different rare earth ions can emit different color tones under different excitation wavelengths, and could be an excellent candidate for a phosphor host. For example, red light emission for Eu^3+^ ion-doped, green light for Tb^3+^ ion, blue light for Tm^3+^ ion, and near white light for Dy^3+^ ion-doped [9,10,11,12].

Rare earth ions have been extensively employed as activators for various phosphors. Sm^3+^ ion is widely used as an activator of reddish orange emission due to its ^4^G_5/2_ → ^6^H_J_ (J = 5/2, 7/2, 9/2, 11/2) transitions, which is the most suitable source for lighting and display from a practical viewpoint [13,14,15,16]. Based on the previous information, the YInGe_2_O_7_ is chosen to be the host, and references concerning the role of the rare-earth Sm^3+^ ion-doped in YInGe_2_O_7_ have not yet been studied. In this investigation, YInGe_2_O_7_ samples doped with different Sm^3+^ concentration phosphors have been prepared by a planetary ball mill solid state reaction process at 1300 °C in air, and the characterization and luminescent properties have also been examined.

## 2. Experimental Procedure

### 2.1. Preparation of Samples

The Sm^3+^ ion-doped YInGe_2_O_7_ phosphor was prepared using a planetary ball mill solid state reaction with Y_2_O_3_, In_2_O_3_, GeO_2_, and Sm_2_O_3_ powders. The starting materials (99.99% purity) were purchased from the Sigma-Aldrich Chemical Company (St. Louis, MO, USA) and Alfa Aesar (Ward Hill, MA, USA). The materials were weighed according to the stoichiometric ratios and mixed by a planetary ball mill (Microwolf PA02, Torrey Hills Technologies, San Diego, CA, USA) at a rotational speed of 150 rpm for 15 min with zirconia balls (Φ = 5 mm, 80 balls) in a polyethylene jar. After mechanically mixing, the mixtures were calcined at 1300 °C in air for 10 h. 

### 2.2. Characterization

The structure for Sm^3+^ ion-doped YInGe_2_O_7_ phosphors were identified by X-ray powder diffractometry (XRD, Rigaku Dmax-33 X-ray diffractometer, Tokyo, Japan) with Cu-Kα radiation with a source voltage of 30 kV and a current of 20 mA. The surface morphology was examined using high-resolution scanning electron microscope (HR-SEM S-4200, Hitachi, Tokyo, Japan). Optical absorption spectra for phosphor powders were placed in a holder, and measured using an ultraviolet-visible (UV–Vis) spectrophotometer (U-3010, Hitachi, Tokyo, Japan) in the range of 200–700 nm at room temperature. Both excitation and luminescence spectra of the phosphors were recorded on a Hitachi F-4500 fluorescence spectrophotometer using a 150 W xenon arc lamp as the excitation source at room temperature.

## 3. Results and Discussion

### 3.1. Structure

**Figure 1:**
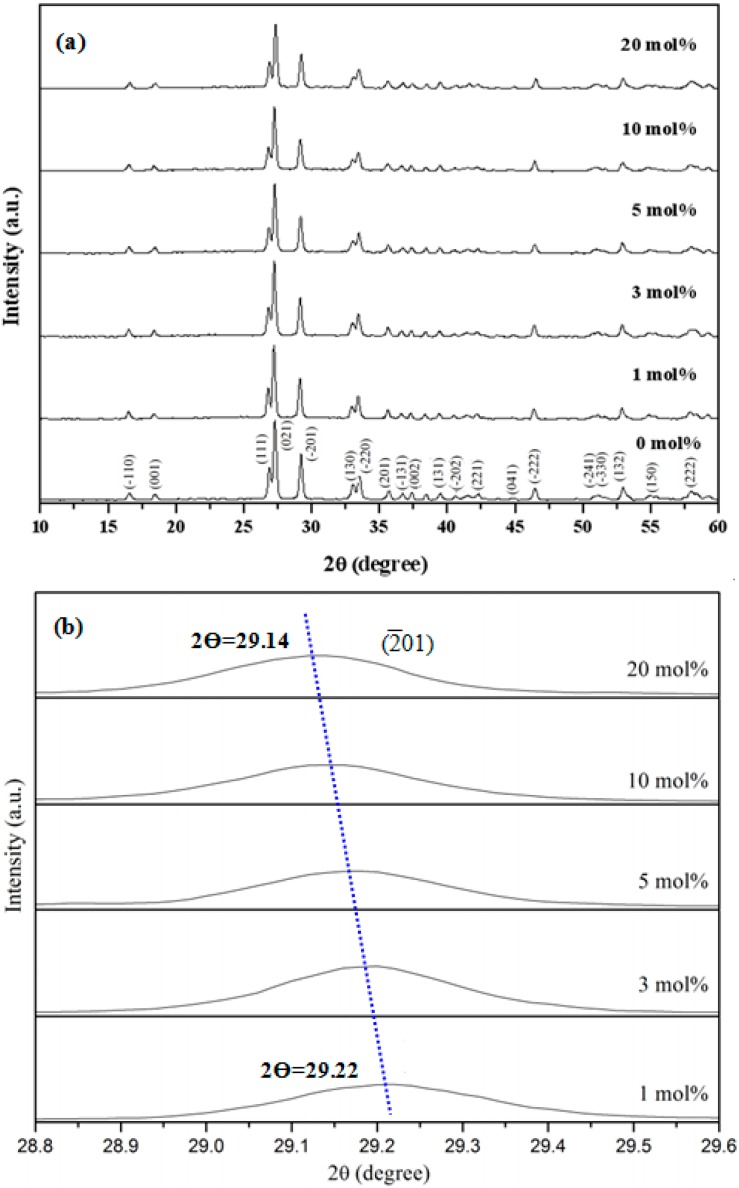
(**a**) X-ray diffraction patterns of YInGe_2_O_7_ doped with different Sm^3+^ ion concentrations calcined at 1300 °C for 10 h in air; (**b**) The peak position of (201) diffraction plane as a function of Sm^3+^ ion concentration.

Figure 1a shows the XRD patterns of YInGe_2_O_7_ doped with different Sm^3+^ ion concentrations. All of the diffraction peaks are attributed to the monoclinic YInGe_2_O_7_ phase. These results demonstrate that the Sm^3+^ ion (r = 0.964 Å) incorporated into the Y_1-x_Sm_x_InGe_2_O_7_ monoclinic structure and replaced the Y^3+^ ion (r = 0.90 Å) to form the solid solution [17]. The full width of half maximum (fwhm) of these peaks seems to increase and the crystallinity of YInGe_2_O_7_:Sm^3+^ becomes worse with the increase in Sm^3+^ ion concentrations. In addition, the diffraction peaks seem to shift to a lower diffraction angle as the Sm^3+^ ion concentration increases. Figure 1b shows the diffraction peaks of the (201) shift to a lower diffraction angle as the Sm^3+^ ion concentration increases. The shift in the position of the peak is Δθ = 0.08° between the Sm^3+^ ion concentrations of Sm^3+^ = 1 mol % and 20 mol %. These results indicate that the lattice constant for the Sm^3+^ ion-doped phosphor is larger than that for the Sm^3+^ free YInGe_2_O_7_ phosphor. Because the Sm^3+^ ion has a larger radius than the Y^3+^ ion, lattice distorts and intra-stress occurs when the concentration of Sm^3+^ ions increases sufficiently to allow the substitution of Y^3+^ ions into the YInGe_2_O_7_ crystal.

### 3.2. Microstructures

**Figure 2:**
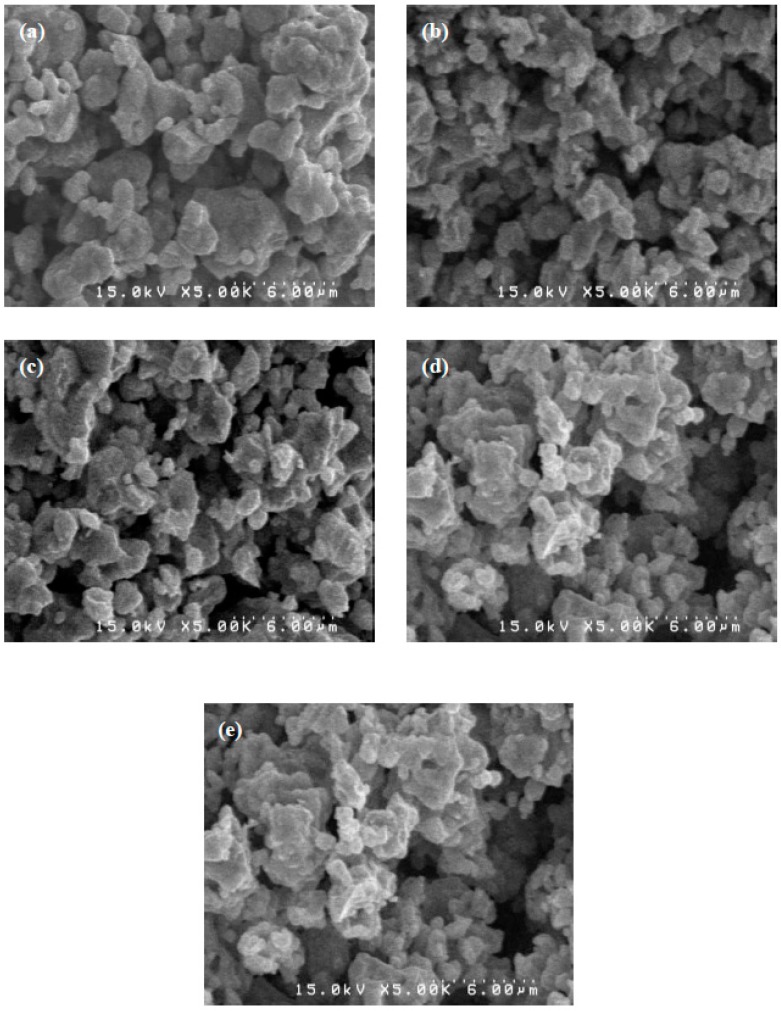
The SEM micrograph of YInGe_2_O_7_ doped with (**a**) 1; (**b**) 3; (**c**) 5; (**d**) 10; and (**e**) 20 mol % Sm^3+^ ions powders calcined at 1300 °C for 10 h in air.

The size and shape of phosphor particles affects the emission intensity and the efficiency of a device. The size of phosphor particles should be as homogeneous as possible without any aggregates or agglomerates, and the surface should also be as smooth as possible and have a high crystallization degree to improve efficiency [17]. Figure 2 shows the SEM micrograph of YInGe_2_O_7_ powders doped with different Sm^3+^ ion concentrations calcined at 1300 °C for 10 h in air. The results show that the crystallized particles aggregated and were irregular. The powder micrographs seem not to obviously vary as the Sm^3+^ ion concentration increases, which indicates that there are no influences on the surface morphology of the powders.

### 3.3. Optical Properties

**Figure 3:**
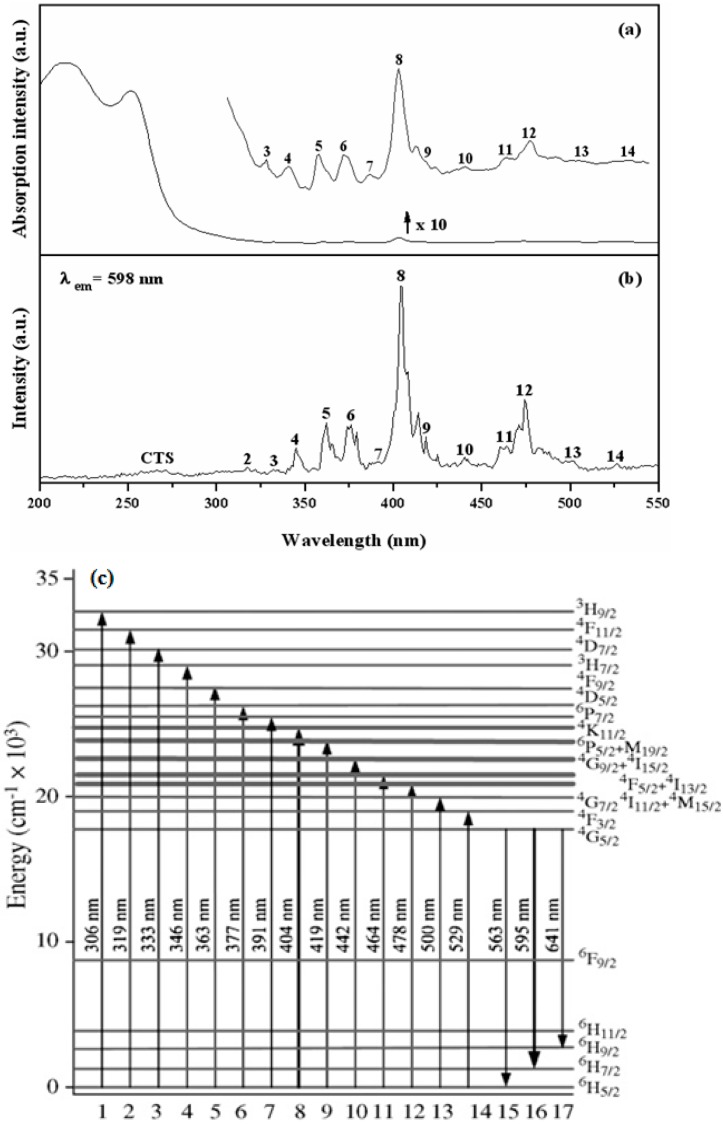
(**a**) Absorption and (**b**) excitation spectra (λ_em_ = 598 nm) for YInGe_2_O_7_ doped with 3 mol % Sm^3+^ ion calcined at 1300 °C for 10 h in air; and (**c**) is the energy level diagram of Sm^3+^ ion.

Figure 3 show the UV-visible absorption spectrum (Figure 3a), and excitation spectrum (λ_em_ = 598 nm) (Figure 3b) for YInGe_2_O_7_ doped with 3 mol % Sm^3+^ ion calcined at 1300 °C for 10 h in air. Figure 3c is the energy level diagram of Sm^3+^ ion. According to the previous study, there are two major absorption bands appearing in the absorption spectrum for pure YInGe_2_O_7_. The first absorption band between 200 and 280 nm can be assigned to the charge transfer between the In^3+^ and O^2−^ ions of the InO_6_ anionic group in the host lattice [17,18]. Another absorption band between 280 and 450 nm is caused by the oxygen deficient center of the GeO_4_ anion [19]. For YInGe_2_O_7_:3 mol % Sm^3+^, the compounds exhibited an absorption peak between 230 to 290 nm due to the charge transfer state (CTS) of the Sm^3+^-O^2−^ ions [20,21], which overlaps with the absorption band of the host lattice and causes the charge transfer state phenomenon to be undetectable in this study.

A series of sharp absorption bands present between 310 and 540 nm, centered at 332, 345, 361, 376, 391, 404, 418, 440, 464, 474, 501, and 526 nm for each absorption peak labeled from 3–14 in the absorption spectrum, respectively, which correspond to the typical f–f transition of Sm^3+^ ions [21,22,23,24]. The strongest peak is located at 404 nm, which can be assigned to the ^6^H_5/2_ → ^4^K_11/2_ transition [24]. The excitation spectrum (λ_em_ = 598 nm) for YInGe_2_O_7_ doped with 3 mol % Sm^3+^ ions calcined at 1300 °C for 10 h in air is shown in Figure 3b. As can be seen, there are two parts for excitation behavior: (1) A weak broad band between 235 and 285 nm is the charge transfer state (CTS) bands due to the samarium–oxygen interactions [20,21]; and (2) A series of excitation peaks in the range from 292 to 490 nm are due to the typical intra-4f transitions of the Sm^3+^ ions that appear at 345, 361, 376, 391, 404, 418, 440, 464, 474, 501, and 526 nm (labeled 4–14 in the excitation spectrum), which were attributed to (^6^H_5/2_ → ^3^H_7/2_), (^6^H_5/2_ → ^4^F_9/2_), (^6^H_5/2_ → ^4^D_5/2_), (^6^H_5/2_ → ^6^P_7/2_), (^6^H_5/2_ → ^4^K_11/2_), (^6^H_5/2_ → ^6^P_5/2_ + ^4^M_19/2_), (^6^H_5/2_ → ^4^G_9/2_ + ^4^I_15/2_), (^6^H_5/2_ → ^4^F_5/2_ + ^4^I_13/2_), (^6^H_5/2_ → ^4^I_11/2_ + ^4^M_15/2_), (^6^H_5/2_ → ^4^G_7/2_), and (^6^H_5/2_ → ^4^F_3/2_) transitions [21,22,23,24]. The strongest excitation peak is located at 404 nm, and it is in good accordance with the absorption analysis results.

**Figure 4:**
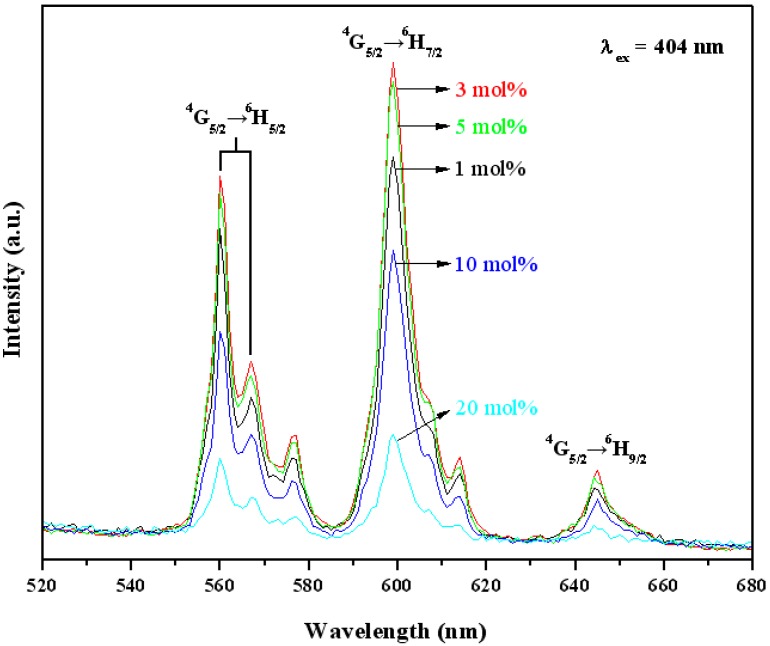
The emission spectra (λ_ex_ = 404 nm) of YInGe_2_O_7_ doped with different concentration of Sm^3+^ ions calcined at 1300 °C for 10 h in air.

Figure 4 shows the photoluminescence emission spectra for YInGe_2_O_7_ doped with different concentrations of Sm^3+^ ions under an excitation wavelength of 404 nm. There are two stronger emission peaks located at 560–570 nm, and 598 nm correspond to the Sm^3+^ intra-4f transition from the excited levels to lower levels, the ^4^G_5/2_ → ^6^H_5/2_ and ^4^G_5/2_ → ^6^H_7/2_ transitions, respectively [18,19]. Another weak emission peak located at 645 nm is due to the ^4^G_5/2_ → ^6^H_9/2_ transition [18]. Due to the absence of a center of symmetry, the 4f orbitals mix with the opposite parity orbitals resulting in the appearance of electric-dipole transitions (^4^G_5/2_ → ^6^H_9/2_) [19], and their intensity is hyper-sensitive to the variation of the local structure environment of the Sm^3+^ ions. While the ^4^G_5/2_ → ^6^H_7/2_ emission is a magnetic-dipole allowed transition, its intensity hardly changes with the local structure symmetry of the Sm^3+^ ions [25]. The higher intensity for the ^4^G_5/2_ → ^6^H_7/2_ transition appears in this study that means the Sm^3+^ ion occupied a symmetry lattice.

In general, fluorescence describes the spontaneous emission of a photon from a molecule or atom after excitation of the electronic system by absorption of light. The emitted photon usually has less energy compared to the excitation photon and consequently has a longer wavelength (Stokes shift) which leads a blue excitation may cause green emission. If the two atoms are close very much, the energy can ‘hop’ directly from the ‘donor’ to the ‘acceptor’, and does not involve the emission and re-absorption of a light. This direct exchange of energy is called Förster Resonance Energy Transfer (FRET) [26]. If FRET occurs, the collected emission is normally not only green but red photons will also be emitted if the sample is illuminated with blue light. Therefore, the green and red light emissions are observed in the emission spectrum for YinGe_2_O_7_:Sm^3+^ phosphor under an excitation wavelength of 404 nm in this study.

According to the studies [8], the In^3+^ and Y^3+^ cations occupy the same octahedral site forming a hexagonal arrangement on the a–b planes of the YInGe_2_O_7_ structure. In their turn, the hexagonal arrangements of InO_6_/YO_6_ octahedral layers are held together by sheets of isolated diorthogroups composed of a double tetrahedron sharing a common vertex, and every two GeO_5_ hexahedrons form the Ge_2_O_9_ structure via oxygen ion sharing which causes the YO_6_ octahedral layer to become disordered and introduce the non-symmetric center of lattice due to internal stress. Therefore, when a Sm^3+^ ion substitutes the Y^3+^ ion lattice position, and it is guessed that the Sm^3+^ ion occupies such a non-symmetric center to cause the intensity of ^4^G_5/2_ → ^6^H_9/2_ transition higher than that of ^4^G_5/2_ → ^6^H_7/2_ transition. However, it is not true in this study, and it might be that the radius of Sm^3+^ ion is larger than the Y^3+^ ion, and leads the Sm^3+^ ion lattice position to become more symmetrical.

**Figure 5:**
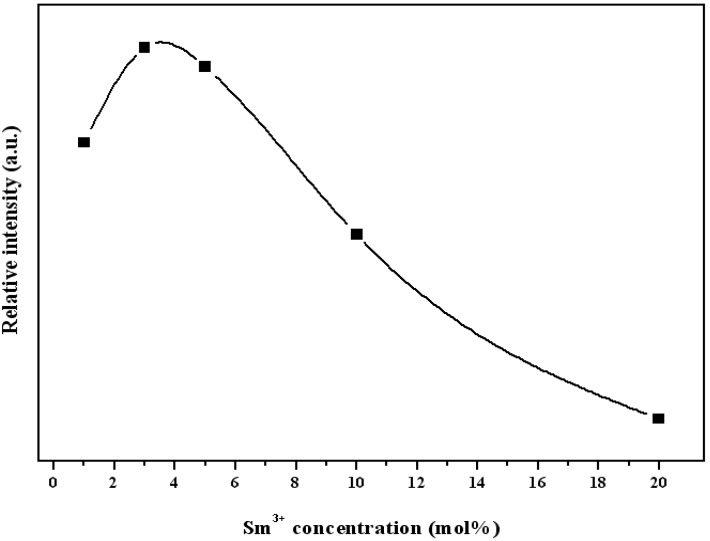
The relationships between the intensity of the emission peak (^4^G_5/2_ → ^6^H_7/2_) and Sm^3+^ ion concentrations.

Figure 5 shows the relationships between e emission intensity of the ^4^G_5/2_ → ^6^H_7/2_ transition of Sm^3+^ ion in YInGe_2_O_7_ plotted versus the Sm^3+^ ion concentrations under an excitation of 404 nm. The emission intensity of ^4^G_5/2_ → ^6^H_7/2_ transition increases with increasing Sm^3+^ ion concentration in the lower Sm^3+^ ion contents region until the saturated PL intensity is reached and then decreases, which indicated that the concentration quenching is active when the Sm^3+^ ion concentrations is higher than 3 mol.%. In addition, the quantum yield (1 mol %:5.5, 3 mol %:10.5, 5 mol %:9.2, 10 mol % 4.7, 20 mol %:1.03) also appears the same tendency for various Sm^3+^ ion-doped phosphor. This is due to the distance between the Sm^3+^ ions becoming shorter, changing the cross-relaxation mechanism to active [17]. The following cross-relaxation may occur

^4^G_5/2_ (Sm I) + ^6^H_5/2_ (Sm II) → ^6^F_9/2_ (Sm I) + ^6^F_9/2_ (Sm II) (1)

**Figure 6:**
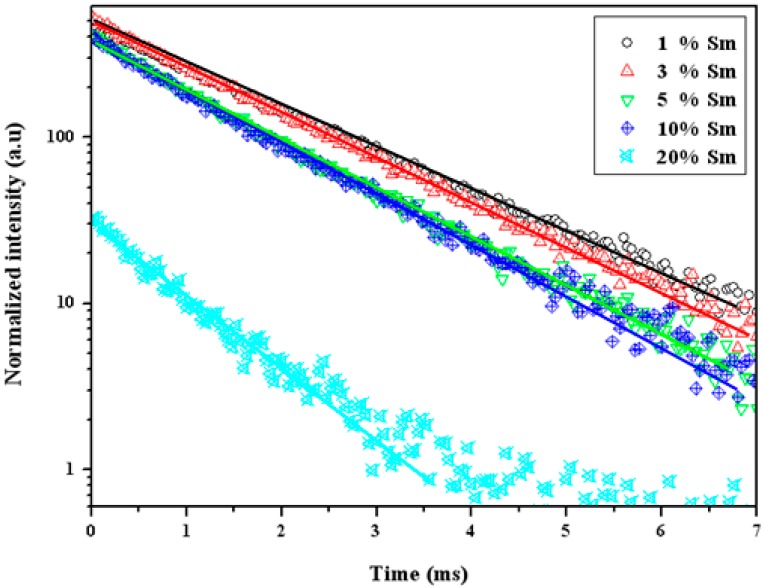
Decay curves for the ^4^G_5/2_ → ^6^H_7/2_ transition of YInGe_2_O_7_ doped with different Sm^3+^ ion concentrations under an excitation of 404 nm.

Figure 6 shows the decay curve and decay time of YInGe_2_O_7_ doped with different Sm^3+^ ion concentrations under an excitation of 404 nm with signals detected at 598 nm. As can be seen, the decay time decreases from 4.5 to 0.8 ms as Sm^3+^ concentration increases from 1 to 20 mol %. At low Sm^3+^ ion concentrations (1 and 3 mol %), the decay rate is almost the same, but decreases rapidly when the Sm^3+^ ion concentrations are higher than 3 mol %. This is caused by the effect of the energy exchange between the Sm^3+^ ions as the distance between the Sm^3+^ ions decreased with increasing Sm^3+^ ion concentrations, enhancing the energy depletion rate and causing the decay time to decrease. Moreover, all of the decay curves were attributed to a single exponential behavior, indicating that the decay mechanism of the ^4^G_5/2_ → ^6^H_7/2_ transition is a single decay component between Sm^3+^ ions only.

**Figure 7:**
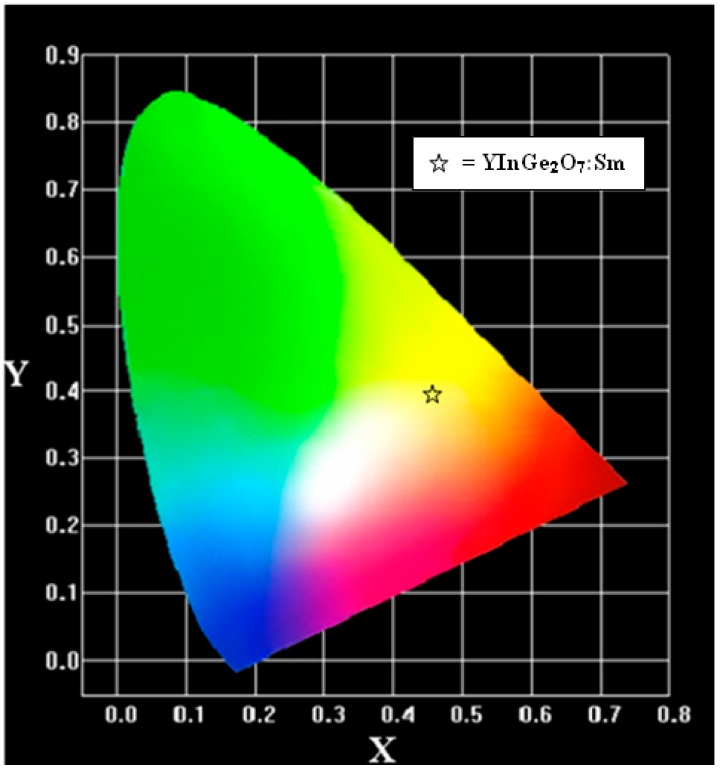
The CIE chromaticity diagram for YInGe_2_O_7_:Sm^3+^ phosphors.

In YInGe_2_O_7_**:**Sm^3+^ system, different concentrations of Sm^3+^ ion did not change the shape of curves but did change the intensities of the emission spectra. Figure 7 shows the CIE color coordinates of YInGe_2_O_7_ doped with 3 mol % Sm^3+^ ions. The color coordinates of the emission were x = 0.457 and y = 0.407, which is located in the orange-yellow region.

## 4. Conclusions

A new phosphor, Sm^3+^ ion-doped YInGe_2_O_7_, was synthesized and its luminescence properties were investigated. The XRD patterns show that all of the peaks can be attributed to the monoclinic YInGe_2_O_7_ crystal structure when the Sm^3+^ ion concentration is increased up to 20 mol %. Under an excitation of 404 nm, there are two stronger emission peaks located at 560–570 nm, and 598 nm corresponding to the Sm^3+^ intra-4f transition for the ^4^G_5/2_ → ^6^H_5/2_ and ^4^G_5/2_ → ^6^H_7/2_ transitions, respectively. Another weak emission peak located at 645 nm is due to the ^4^G_5/2_ → ^6^H_9/2_ transition. The concentration quenching effect occurs when Sm^3+^ ions rise above 3 mol % with a CIE color coordinate of x = 0.457 and y = 0.407, which is located in the orange-yellow light region. The decay curve results indicate that the decay time decreases from 4.5 to 0.8 ms as Sm^3+^ concentration increases from 1 to 20 mol %, and the decay mechanism of the ^4^G_5/2_ → ^6^H_7/2_ transition is a single decay component between Sm^3+^ ions only.
